# The impact of ivermectin on onchocerciasis in villages co-endemic for lymphatic filariasis in an area of onchocerciasis recrudescence in Burkina Faso

**DOI:** 10.1371/journal.pntd.0009117

**Published:** 2021-03-01

**Authors:** Achille S. Nikièma, Lassane Koala, Apoline K. Sondo, Rory J. Post, Alain B. Paré, Claude M. Kafando, Roger S. Kambiré, Bazoumana Sow, Clarisse Bougouma, Roch K. Dabiré, Soungalo Traoré

**Affiliations:** 1 Institut de Recherche en Sciences de la Santé (IRSS), Direction Régionale de l’Ouest, Bobo Dioulasso, Burkina Faso; 2 Université Ouaga I Pr Joseph ki-Zerbo, Unité de Formation et de Recherche en Sciences de la Santé, Ouagadougou, Burkina Faso; 3 Disease Control Department, London School of Hygiene & Tropical Medicine, London, United Kingdom; 4 School of Biological & Environmental Sciences, Liverpool John Moores University, Liverpool, United Kingdom; 5 Ministère de la Santé, Direction de la Protection de la Santé de la Population, Programme National lutte contre les Maladies Tropicales Négligées, Ouagadougou, Burkina Faso; 6 Medical Entomologist (Freelance), Ouagadougou, Burkina Faso; Watford General Hospital, UNITED KINGDOM

## Abstract

In Burkina Faso, onchocerciasis was no longer a public health problem when the WHO Onchocerciasis Control Programme in West Africa closed at the end in 2002. However, epidemiological surveillance carried out from November 2010 to February of 2011, showed a recrudescence of infection in the Cascades Region. This finding was made at a time when ivermectin, a drug recommended for the treatment of both onchocerciasis and lymphatic filariasis, had been distributed in this area since 2004 for the elimination of lymphatic filariasis. It was surprising that ivermectin distributed for treating lymphatic filariasis had not prevented the recrudescence of onchocerciasis. Faced with this situation, the aim of our study was to evaluate the effectiveness of ivermectin on the onchocerciasis parasite. The percentage reduction in microfilarial load after treatment with ivermectin was used as a proxy measure for assessing possible resistance. A cohort study was carried out with 130 individuals who had tested positive for microfilariae of *Onchocerca volvulus* in 2010 using microscopic examination of skin-snip biopsies from five endemic villages. Subjects were followed from July 2011 to June 2012. The microfilarial load of each individual was enumerated by skin-snip biopsy in 2010, prior to the first ivermectin treatment against onchocerciasis under community guidelines. All individuals received two ivermectin treatments six months apart. In 2012, the microfilarial loads were determined again, six months after the second round of ivermectin and the reductions in parasite loads were calculated to measure the impact of the drug. The percentage reduction of the microfilarial loads ranged from 87% to 98% in the villages. In all villages, there was a statistically significant difference between the average microfilarial loads in 2010 and 2012. The level of reduction of microfilarial loads suggests that ivermectin is effective against the recrudescent population of *O*. *volvulus* in Cascades Region of Burkina Faso. Further investigations would be necessary to determine the causes of the recrudescence of onchocerciasis. (For French language abstract, see S1 Alternative Language Abstract—Translation of the Abstract into French by the authors.)

## Author summary

In 1989, onchocerciasis had been eliminated as a public health problem throughout Burkina Faso by insecticidal treatment of vector breeding sites, but epidemiological surveys along the Comoé River in 2010/11 revealed a recrudescence of infection rates. Modern onchocerciasis control is based upon mass drug administration using ivermectin, and hence biannual distribution of ivermectin was instigated to bring the recrudescence under control. However, it was by no means certain that this was an appropriate strategy because the area was already under mass drug administration with ivermectin since 2004 to eliminate lymphatic filariasis. *Onchocerca volvulus* adult females with reduced susceptibility to ivermectin have been reported from Ghana, and if the Burkinabe recrudescence was the result of reduced susceptibility, ivermectin might fail to solve the problem. To test this, a cohort of positive people was examined for the density of microfilariae in their skin six months after the second round of ivermectin. The reduction in microfilarial load was substantial, and comparable to levels found in ivermectin-naïve populations (normally assumed to be susceptible), but greater than Ghanaian populations with reduced susceptibility. On this basis we conclude that the Burkinabe recrudescence was not the result of parasites with reduced susceptibility to ivermectin, and biannual treatment remains the chosen means to deal with the recrudescence.

## Introduction

Onchocerciasis, or river blindness, is a severely debilitating vector-borne parasitic disease which has been targeted for elimination by the World Health Organization (WHO) by 2025 [1]. It is caused by infection by the filarial nematode worm, *Onchocerca volvulus*, which is transmitted from person to person by blood-feeding blackflies of the genus *Simulium* (Diptera: Simuliidae). Clinical manifestations of infection include visual impairment (including blindness), skin disease (sub-cutaneous nodules, itching, loss of elasticity and depigmentation) and epilepsy, and as such onchocerciasis is a significant cause of poverty. WHO estimates that 205 million people live in endemic areas worldwide, of whom, more than 99% are in Africa [1,2]. Until the late 1980s there were only a few filaricidal drugs which could be used to treat onchocerciasis and they all had severe side effects. The only means to control onchocerciasis in the community was through vector control, and this was applied from 1974 to 2002 by the WHO Onchocerciasis Control Programme (OCP), which included all endemic areas in Burkina Faso. By 1986 prevalence of microfilariae was less than 5% in all villages in the Comoé river valley in Burkina Faso Cascades Region (except for two, which subsequently dropped to 0% and 3.7% by 1999). OCP ceased vector control operations in this region in 1989 because onchocerciasis was judged to have been reduced to insignificant levels (i.e. less than 5%). However, in 2001 the prevalence in one village in the Comoé river valley had increased to 39.6%, and two more had increased above 5% by 2007. New epidemiological surveys from November 2010 to February 2011 showed that in 13 out of 30 villages in the Comoé river valley prevalence of microfilaremia was above 5% [3]. The reason for the recrudescence is unclear, but it was associated with high levels of vector infectivity [4] and did not appear to be happening in other parts of southern Burkina Faso [5]. It was particularly worrying because it happened in an area where ivermectin, had been distributed by Mass Drug Administration (MDA) once a year since 2004 for the elimination of lymphatic filariasis (LF) (i.e. for six consecutive years up to 2010). Ivermectin was registered for use against onchocerciasis in 1987, and remains the only drug recommended for the treatment of onchocerciasis through MDA [6], and therefore biannual MDA with ivermectin was introduced to try to bring the recrudescence under control [3].

The main effects of ivermectin are to kill microfilariae and temporarily sterilise the adult female parasite. It was first introduced for the control of onchocerciasis in Africa, but was used successfully for elimination of transmission by the Onchocerciasis Elimination Programme for the Americas (OEPA), and further studies in Africa also showed that onchocerciasis could be eliminated through Community Directed Treatment with Ivermectin (CDTI—a form of MDA) after about 15 years in endemic communities [7,8,9]. Consequently, WHO shifted its objective from control of onchocerciasis as a disease of public health importance to elimination of transmission throughout Africa using CDTI [10]. However, it is potentially problematic that the elimination programme is based on a single suitable drug because of the ever-present threat that the parasite might evolve resistance, and the persistence of microfilaremia was already observed in a number of communities in Ghana after multiple ivermectin treatments [11], and the lack of response in this part of Ghana had persisted up to 2010, and possibly beyond [12].

Considering the high prevalence of onchocerciasis recorded in the 2010/11 survey [3], it seems that the years of ivermectin treatments carried out to eliminate LF have had little or no effect on *O*. *volvulus*. This situation raised the question whether ivermectin was ineffective on these parasites, and this study aims to test this by presenting the observed changes in microfilarial load in people from five endemic villages in the Cascades Region who tested positive on skin-snip biopsy and took two ivermectin treatments six months apart. The percentage reduction in microfilarial load after ivermectin treatment is used as a proxy measure for assessing possible resistance.

## Methods

### Ethics statement

The National Onchocerciasis Control Programme received blanket approval from the Ethics Committee of the Ministry of Health in 2009 for carrying out the routine activities for the control and elimination of onchocerciasis (including epidemiological evaluation by skin-biopsy as carried out for this study). In the field on the day of the survey, the team organised an awareness meeting with the village population about the disease and its diagnosis using skin snips. The study was explained to members of the selected communities (in their own language), and those who agreed to participate were selected after free, oral and informed consent in the case of individuals aged 18 and over, and the consent of parents or guardians in the case of those under the age of 18.

### Study area and village selection

The study was conducted in the Cascades Health Region, along the Comoé River (which rises in Burkina Faso and flows southwards through Côte d’Ivoire and into the sea). This region is located in the southwest of the country and is irrigated by two rivers (the Comoé and the Léraba) and their tributaries. The region has two seasons, a rainy season from April to October and a dry season throughout the rest of the year. The type of vegetation found in the area is guinea savannah, where vector blackflies of the *Simulium damnosum* complex are generally abundant during the rainy season [13,14]. However, a dam with a capacity of about 38 million cubic meters was built across the River Comoé upstream, near the village of Moussodougou, in 1989, and this dam ensures sufficient flow for the production of blackflies in the rapids located in the study area during the dry season. The Cascades Health Region is subdivided into three Health Districts, Banfora (6295 km^2^), Mangodara (2808 km^2^) and Sindou (9302 km^2^), and the study villages are located in the health districts of Banfora (4 villages) and Mangodara (1 village).

**Fig 1:**
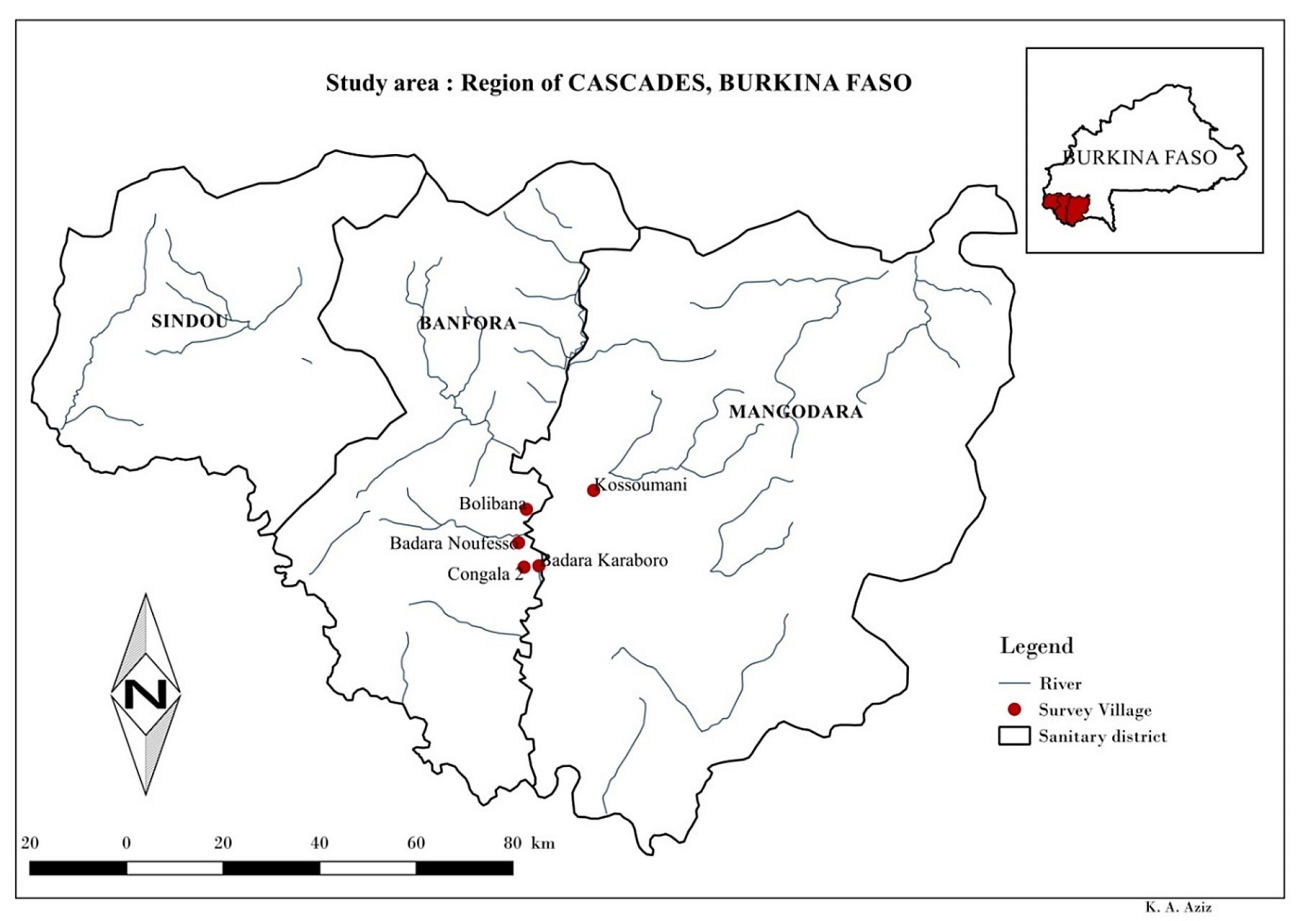
Geographic Location of Study Sites in the Cascades Health Region and Health Districts (= Sanitary Districts) in Burkina Faso.

Five villages in the Cascades Region: Badara Karaboro, Badara Nofesso, Bolibana, Congala 2, Kossoumani (Fig 1) were selected for the study. These villages were amongst the worst affected by the recrudescence with unstandardised microfilarial prevalences ranging from 26% to 71% during the 2010 onchocerciasis epidemiological assessment surveys [3]. They are all located between 200 metres (Badara Karaboro) and 2 kilometres (Kossoumani) on either side of the Comoé River. Villages with high prevalences were chosen because it is in these villages that any possible insensitivity of the parasites to ivermectin will be easiest to detect, and also these sorts of villages are most at risk because dermatological and ocular lesions are likely to appear early [15].

### Study design

A cohort study of individuals who tested microfilarial positive by skin snip biopsy was established from each of the five study villages. The microfilarial load of each participant was determined by skin-snip biopsy in 2010 before the first round of CDTI in 2011. CDTI was carried out as recommended by the WHO African Programme for Onchocerciasis Control (APOC) [16]. The ivermectin treatment protocol was a single dose of 150 μg/kg body weight at six-month intervals. The effect of two doses of ivermectin on microfilarial load was estimated by skin-snip six months after the second dose.

### Study population

The study population was made up of all the positives from the five villages, making a total of 231 diagnosed individuals. They were followed for 12 months between 2011 and 2012. Their microfilarial load was measured in April 2010, before the first dose of ivermectin (July 2011). The microfilarial load was again measured a second time in June 2012 (six months after the second dose of ivermectin, which was in December 2011, and just before the third dose of ivermectin), from individuals who had taken both ivermectin treatments during the CDTI treatment campaigns (i.e. both in July and December 2011) and who were present on the day of the survey. Of the original 231 people who were skin-snip positive, 101 failed to meet the subsequent requirements of the study for various reasons, and the final size of the cohort was 130 people. The reasons for the 101 people failing to complete the course were varied and included, emigration, death, failure to take one or both doses of ivermectin (as a result of pregnancy or absence at the time of drug distribution) and absence at the time of the final skin-snipping.

### Parasitological diagnostic method

The skin-snip biopsy method was used as the diagnostic method according to WHO standards [17]. Skin snips were taken from the right and left iliac crests using a 2-mm Holth corneoscleral punch. These skin snips were then incubated in distilled water for 30 minutes and enumeration of microfilaria was carried out on site. Negative skin ships were then incubated in physiological saline and re-examined 24 hours later. No further skin snips were positive after 24 hours and therefore only results after 30 minutes were used in the analyses of data.

### Data analysis

The arithmetic mean of the microfilarial load and the percentage reduction of the arithmetic mean of the microfilarial load were determined. Software R version 3.5.3 was used for the statistical analyses. A Student t Test were used to compare mean microfilarial loads between 2010 and 2012 with a statistical significance level lower than 5%.

Microfilarial load percentage reduction = (C1-C2)/C1*100

C1 = microfilarial load before and C2 = microfilarial load after

## Results

The full data set is presented in Supporting Information S1 Table. A total of 130 individuals were examined by skin snip biopsy in 2012, of whom 45% were females. The age extremes were 5 years and 70 years. The maximum individual mean microfilarial load was 115 mf/b (microfilariae per biopsy) in 2010 and 26 mf/b in 2012. The microfilarial loads of 101 people who were skin snip positive in 2010, but dropped out of the study cohort is given in Supporting Information S2 Table.

In comparison with 2010, the microfilarial load was significantly reduced in all villages in 2012 after two treatments with ivermectin (Table 1). In 2012, the mean village load ranged from 0.045 to 2.021 microfilariae per biopsy. Both before and after CDTI, the lowest microfilarial loads were recorded in the village of Kossoumani and the highest load in Bolibana. The percentage reduction of the mean load varied from 87.1% to 97.9%. The percentage reduction of the microfilarial load was higher in Kossoumani than in the other villages. In both males and females, there was a statistically significant difference between the average loads in 2012 compared to 2010. However, there was no significant difference in the reduction between males and females.

**Table 1:** Microfilarial Load of Participants in 2010 and 2012 by Village and by Sex.

Variables (Village & Sex)	Number examined	2010 Microfilarial load mf/b (sd)	2012 Microfilarial load mf/b (sd)	Reduction (%)	p value
1a. By village					
Badara Karaboro	20	17.425 (16.784)	1.275 (2.658)	92.68	0.0003
Badara Nofesso	26	18.615 (24.722)	1.230 (1.893)	93.39	0.0009
Bolibana	47	18.638 (26.487)	2.021 (4.798)	89.15	0.0004
Congala 2	26	11.346 (8.902)	1.461 (3.042)	87.12	0.00002
Kossoumani	11	2.136 (2.335)	0.045 (0.150)	97.89	0.014
1b. By sex					
Males	72	14.791 (20.720)	1.569 (3.966)	89.39	
Females	58	16.582 (21.720)	1.344 (2.761)	91.89	
Total	130	-			

**Notes:** mf/b = mean number of microfilariae per biopsy; sd = Standard deviation.

In all age groups, there was a statistically significant difference between the average loads in 2012 compared to 2010 (Table 2). However, there was no significant difference in the reduction between age groups, although the reduction in the microfilarial load was highest in the 50+ age group and lowest in the 20–34 age group.

**Table 2:** Microfilarial Load of Participants in 2010 and 2012 by Age Group.

Age group (year)	Number examined	2010 Microfilarial load mf/b (sd)	2012 Microfilarial load mf/b (sd)	Reduction (%)
5–19	60	14.567 (21.248)	1.576 (4.382)	89.18
20–34	31	13.274 (15.610)	1.645 (2.872)	87.60
35–49	24	18.708 (23.190)	1.666 (2.685)	91.09
50+	15	20.400 (28.351)	0.433 (0.677)	97.87
Total	130			

**Notes:** mf/b = mean number of microfilariae per biopsy; sd = Standard deviation.

## Discussion

This study was carried out to monitor the response of the recrudescent population of *O*. *volvulus* to ivermectin treatment which was introduced to eliminate the recrudescence. The recrudescence had taken place in spite of MDA using ivermectin against LF, and it was possible that the recrudescence was enabled by parasite resistance to ivermectin. It is unlikely that any resistance might have been the result of immigration of infected humans carrying ivermectin-tolerant parasites from Ghana (see above) because previous investigations have shown that human infections were not linked to immigration status in the Burkina Faso recrudescence [5], but resistant parasites might still have been carried into the area by migrant vectors, or resistance might have evolved independently in Burkina Faso. This study on the change in the microfilarial load after one year of biannual treatment with ivermectin along the Comoé River was the first of its kind in Cascades Region. Monitoring of the microfilarial load has been done in the past, but as part of the evaluation of the impact of vector control on the epidemiology of onchocerciasis [3].

In this study, evaluation of the effectiveness of ivermectin in reducing microfilarial loads in treated, positive individuals after two rounds of CDTI yielded percentage reductions in all five communities of 87–98% (mean 92%). There have been only four previous studies which recorded the percentage reduction after the second of two biannual treatments, and they recorded the percentage reductions in Liberia (93.8%) [18], Guatemala (mean of 72.2% across three communities) [19] and Sierra Leone (95%) [20]. These reductions were measured in populations which had not been previously exposed to ivermectin and were presumed to be fully susceptible, and our results are comparable. However, in Ghana three communities which had been subject to many annual treatments and were identified as responding poorly to ivermectin (and possibly showing some form of resistance in the parasites) had also been assessed for response to biannual treatment [12]. They showed reductions of 31% (Asubende) 34% (Kyingakrom) and 69% (New Longoro) amongst mf-positive people (mean of three villages = 44.7%). These reductions are clearly much less than those seen from Cascades Region in Burkina Faso and from the ivermectin-naïve communities elsewhere. These comparisons strongly suggests that the recrudescence of onchocerciasis along the Comoé River is not linked to any ineffectiveness of ivermectin against the *O*. *volvulus* parasite, and therefore ivermectin can continue to be administered to communities to bring the recrudescence under control and take it to elimination. However, it should be noted that these various studies did not all use the same length of time for incubation of skin snips prior to counting of emergent microfilariae. Our study used the standard method which was adopted by WHO more than 50 years ago [17] but is still being used by WHO [21] and is recommended by WHO along with OV-16 serology for routine monitoring and evaluation surveys [22], with counting of emergent microfilariae after 30 minutes. This was also used in Sierra Leone [20], but the Liberian [18] and Guatemalan [19] studies incubated skin snips “overnight” and for 24 hours respectively. The study of the Ghanaian communities [12] were also incubated for 24 hours. Whilst incubation for only 30 minutes is likely to give an underestimate of community microfilarial load (CMFL) compared with longer periods of incubation, there is no evidence that this will distort the relative reduction in the numbers of microfilariae measured in these studies. In any case, our results are more similar to the results from all three studies of ivermectin-naïve communities, and whilst the reductions of microfilariae in Guatemala are a little less (mean reduction of 72.2%), they are still closer to our results (mean reduction of 92%) than they are to the Ghana results (mean reduction of 44.7%).

The factors which allowed the recrudescence to take place have been discussed in depth by Koala et al. [3], but they are still not fully understood. There are two main questions. Firstly, were the recrudescent parasites the offspring of survivors from the pre-OCP population which had not been eliminated, or were they immigrants carried into the area by infected humans or migrant vectors? Secondly, how could the recrudescence have occurred in the face of mass drug administration of ivermectin (against LF)? When the OCP ceased vector control in 1989, the vectors re-established breeding populations more or less immediately. OCP continued to monitor the annual transmission potential for two years and it never reached zero, but it stayed below the threshold recognised by WHO for self-sustaining transmission [10,22]. However, this was not the case for the epidemiological data provided by skin-snips. WHO no longer considers skin snips to be sufficiently sensitive for the assessment of elimination of transmission although they can be used as a tool to monitor progress towards elimination [22]. However, APOC [10] had considered that the epidemiological transmission threshold had been reached when human prevalence of infection was below 5% in all villages and below 1% in 90% of villages, and communities in the Comoé valley in Burkina Faso had not reached this threshold when vector control ceased, having 11% of communities above 5% prevalence and 58% of communities above 1% prevalence [3]. Whilst this evidence is far from conclusive, it certainly indicates the possibility that a local population of parasites survived the years of vector control, and the possibility of parasite immigration carried by either vectors or humans appears less likely. This is because the adjacent Leraba River valley was subject to higher levels of vector immigration than the Comoé valley [3] but was not part of the recrudescence, and infections in the recrudescent area seem to be associated with the resident population rather than those with a migration history [5]. Whether the recrudescent parasite population consisted of local survivors or immigrants, the question still remains as to why it was able to recrudesce in the face of on-going ivermectin distribution against LF. We have now shown that the recrudescent parasites do not seem to be resistant to ivermectin, and so the most likely explanation is an inadequacy of the MDA activities in the Cascades Region by the National Programme for the Eradication of LF. Koala et al. [3] mentioned that during investigations “it became evident that therapeutic coverage had often fallen short. Inhabitants gave a number of reasons for non-participation, including the undesirable effects of the drugs … and a perception that LF was not a grave problem. Furthermore, the programme achieved only weak geographic coverage in these zones (with certain villages untreated)” (see S3 Table), and Nikièma [23] found that the implementation of the CDTI strategy against onchocerciasis in 2011 was better monitored through the treatment register and yielded reliable, valid and satisfactory therapeutic and geographical coverage rates. In conclusion, it seems most likely that a local population of parasites had survived the years of vector control and were able to recrudesce in the face of mass drug administration of ivermectin against LF because the coverage was inadequate. However, further investigations would be needed to determine the causes of the recrudescence with any certainty.

Whilst other studies have shown tolerance (or even resistance) of ivermectin by *O*. *volvulus* in Ghana [24], our results indicate that this was not the case in Burkina Faso, but the recrudescence was still dangerous. Our results show that in 2010, the mean of the microfilarial load was greater than 2 microfilariae/biopsy (mf/b) in all the five villages, and it has been shown that *S*. *damnosum* s.l. vectors were able to ensure the transmission of the parasite in forest regions of Côte d’Ivoire at 1 mf/b [25]. These positive individuals would therefore constitute a risk to the rest of the community. Furthermore, the parasite strain in the area is expected to be the savannah strain (which causes high rates of blindness), and the vectors are expected to belong to the ‘savannah group’ of *S*. *damnosum* s.l. cytospecies which are effective vectors of blinding onchocerciasis [4,26,27].

The reduction of the microfilarial load brought about by CDTI is expected to have an impact on the transmission in the villages. The numbers of microfilaria taken up by the blackflies during their blood-meal is reduced with the corollary that fewer microfilaria can cross the peritrophic membrane to develop into infective larvae [26,28]. It is the infective larvae that are transmitted by the blackfly when it takes its blood-meal. Studies carried out during an outbreak in the Guinea savannah in Ghana showed a 65–85% reduction in transmission of the parasite by savannah species of the *S*. *damnosum* complex during the three months following ivermectin mass distribution [29]. Other data remains to be collected in our study, specifically entomological data, but we could expect that biannual CDTI will reduce the transmission of onchocerciasis in the Comoé valley. Finally, the reduction of the individual microfilarial load from a maximum of 115 mf/b in 2010 to a maximum of 26 mf/b in 2012 should be of benefit to the individual patient. This individual load reduction is characterized as a "curative effect" by some authors [30]. This study did not investigate the dermatological and ophthalmological aspects of onchocerciasis, but the improvement of important symptoms (skin and eye lesions) after administration of ivermectin in onchocerciasis endemic areas in sub-Saharan Africa has been widely reported [31,32,33]. The age group of 50 and over had the highest percentage reduction of microfilariae after ivermectin administration in Cameroon [34]. This might be partly explained by older individuals being carriers of older adult female macrofilariae, which would release fewer microfilariae after ivermectin treatment, as opposed to younger individuals who would be carriers of younger female macrofilariae, which are able to produce more microfilaria after ivermectin treatment.

The effectiveness of ivermectin observed on the parasite in this study suggested that by continuing the CDTI in the region (and maintaining treatment coverage of 80% or higher, with an emphasis on close supervision by health professional and community distributors), it should be possible to eliminate transmission and all the consequent benefits would be felt by the population, as demonstrated by previous studies showing that eliminating onchocerciasis is feasible through regular treatment with ivermectin [7,8,9]. Therefore, the biannual treatments with ivermectin introduced in 2011 have been continued. An epidemiological (skin-snip) survey carried out in 2016 indicated a mean reduction of mf prevalence of around 75% and a second survey is planned for 2021, and the results will be published when the recrudescence is eliminated.

## Supporting information

S1 Alternative Language Abstract Translation of the Abstract into French by the authors. (PDF)

S1 Table Numbers of microfilariae for each iliac crest (mfs/b) in all participants in 2010 and 2012. (PDF)

S2 Table Numbers of microfilariae for each iliac crest (mfs/b) in the 101 people who were skin snip positive in 2010 but failed to complete the cohort requirements to participate in the skin snip assessment in 2012. (PDF)

S3 Table Therapeutic coverage of mass drug administration with ivermectin against lymphatic filariasis in the onchocerciasis recrudescence area of Cascades Region in Burkina Faso up to 2011. (PDF)
