# Supplementary material for: The impact of ivermectin on onchocerciasis in villages co-endemic for lymphatic filariasis in an area of onchocerciasis recrudescence in Burkina Faso
Source: PLoS Negl Trop Dis. 2021 Mar 1;15(3):e0009117. doi: 10.1371/journal.pntd.0009117 (PMC7920372; doi:10.1371/journal.pntd.0009117)
Supplement: S1 Alternative Language Abstract — (PDF) [file pntd.0009117.s001.pdf]

SUPPORTING INFORMATION
Nikiema et al. The impact of ivermectin in an area of onchocerciasis recrudescence.

French Language Abstract.

Title: L'impact de l'ivermectine sur l'onchocercose dans les villages co-endémiques pour la filariose lymphatique dans une zone de recrudescence de l'onchocercose au Burkina Faso

RÉSUMÉ
Au Burkina Faso, l'onchocercose ne constituait plus un problème de santé publique à la fermeture du Programme de Lutte contre l'Onchocercose en Afrique de l'Ouest en 2002. Cependant, la surveillance épidémiologique menée de novembre 2010 à février 2011 a montré une recrudescence de l'infection dans la Région des Cascades. Ce constat a été fait à un moment où l'ivermectine, un médicament recommandé pour le traitement de l'onchocercose et de la filariose lymphatique, était distribué dans cette région depuis 2004 pour l'élimination de la filariose lymphatique. Il est surprenant que l'ivermectine distribuée pour le traitement de la filariose lymphatique n'ait pas empêché la recrudescence de l'onchocercose. Face à cette situation, le but de notre étude était d'évaluer l'efficacité de l'ivermectine sur le parasite de l'onchocercose. Le pourcentage de réduction de la charge microfilarienne après le traitement à l'ivermectine a été utilisé comme mesure de substitution pour évaluer une éventuelle résistance. Une étude de cohorte a été réalisée avec 130 individus qui avaient été diagnostiqués positifs pour les microfilaires d'*Onchocerca volvulus* en 2010 par l'examen microscopique de biopsies cutanées exsangues dans cinq villages endémiques. Les individus ont été suivis de juillet 2011 à juin 2012. La charge microfilarienne de chaque individu a été déterminée par biopsie cutanée exsangue en 2010, avant le premier traitement à l'ivermectine sous directives communautaires contre l'onchocercose. Tous les individus ont reçu deux traitements à l'ivermectine espacés de six mois. En 2012, les charges microfilariennes ont été déterminées à nouveau six mois après le deuxième traitement à l'ivermectine sous directives communautaires et la réduction des charges parasitaires a été calculée pour mesurer l'impact du médicament. Le pourcentage de réduction des charges microfilariennes moyennes variait de 87 % à 98 % dans les villages. Dans tous les villages, il y avait une différence statistiquement significative entre les charges microfilariennes moyennes en 2010 et 2012. Le niveau de réduction des charges microfilariennes suggère que l'ivermectine est efficace contre le parasite *O. volvulus* présent dans la population de la zone de recrudescence dans la région des Cascades au Burkina Faso. Des recherches supplémentaires seraient nécessaires pour déterminer les causes de la recrudescence de l'onchocercose.
