# Supplementary material for: The impact of ivermectin on onchocerciasis in villages co-endemic for lymphatic filariasis in an area of onchocerciasis recrudescence in Burkina Faso
Source: PLoS Negl Trop Dis. 2021 Mar 1;15(3):e0009117. doi: 10.1371/journal.pntd.0009117 (PMC7920372; doi:10.1371/journal.pntd.0009117)
Supplement: S1 Table — (PDF) [file pntd.0009117.s002.pdf]

## SUPPLEMENTARY MATERIAL

Nikiema et al. The impact of ivermectin in an area of onchocerciasis recrudescence.

### S1 Table. Numbers of microfilariae for each iliac crest (mfs/b) in all participants in 2010 and 2012.

| | VILLAGE | SEX | AGE | 2010 ICL | 2010 ICR | 2012 ICL | 2012 ICR |
|---|---|---|---|---|---|---|---|
| 1 | BADARA KARABORO | M | 30 | 40 | 0 | 0 | 2 |
| 2 | BADARA KARABORO | F | 50 | 15 | 13 | 0 | 0 |
| 3 | BADARA KARABORO | M | 20 | 17 | 12 | 4 | 0 |
| 4 | BADARA KARABORO | M | 47 | 100 | 50 | 4 | 1 |
| 5 | BADARA KARABORO | M | 15 | 50 | 30 | 1 | 0 |
| 6 | BADARA KARABORO | M | 17 | 20 | 12 | 0 | 1 |
| 7 | BADARA KARABORO | M | 38 | 15 | 22 | 4 | 4 |
| 8 | BADARA KARABORO | F | 31 | 2 | 2 | 0 | 0 |
| 9 | BADARA KARABORO | M | 8 | 1 | 1 | 0 | 0 |
| 10 | BADARA KARABORO | M | 52 | 10 | 13 | 0 | 0 |
| 11 | BADARA KARABORO | F | 39 | 2 | 1 | 0 | 0 |
| 12 | BADARA KARABORO | F | 5 | 35 | 30 | 0 | 1 |
| 13 | BADARA KARABORO | F | 7 | 22 | 17 | 0 | 1 |
| 14 | BADARA KARABORO | F | 20 | 5 | 15 | 0 | 0 |
| 15 | BADARA KARABORO | M | 12 | 2 | 10 | 4 | 1 |
| 16 | BADARA KARABORO | F | 10 | 26 | 12 | 13 | 10 |
| 17 | BADARA KARABORO | M | 6 | 10 | 15 | 0 | 0 |
| 18 | BADARA KARABORO | M | 18 | 25 | 14 | 0 | 0 |
| 19 | BADARA KARABORO | F | 7 | 3 | 1 | 0 | 0 |

| | | | | | | | |
|---|---|---|---|---|---|---|---|
| 20 | BADARA KARABORO | M | 32 | 12 | 15 | 0 | 0 |
| 1 | BADARA NOFESSO | F | 37 | 10 | 15 | 0 | 0 |
| 2 | BADARA NOFESSO | M | 39 | 15 | 7 | 1 | 0 |
| 3 | BADARA NOFESSO | M | 45 | 80 | 70 | 2 | 0 |
| 4 | BADARA NOFESSO | F | 10 | 60 | 40 | 0 | 0 |
| 5 | BADARA NOFESSO | M | 8 | 120 | 50 | 6 | 10 |
| 6 | BADARA NOFESSO | M | 6 | 10 | 15 | 0 | 0 |
| 7 | BADARA NOFESSO | M | 6 | 12 | 6 | 0 | 4 |
| 8 | BADARA NOFESSO | M | 58 | 12 | 15 | 0 | 2 |
| 9 | BADARA NOFESSO | M | 8 | 17 | 22 | 0 | 1 |
| 10 | BADARA NOFESSO | M | 18 | 10 | 3 | 1 | 2 |
| 11 | BADARA NOFESSO | F | 13 | 20 | 10 | 4 | 0 |
| 12 | BADARA NOFESSO | M | 40 | 5 | 6 | 3 | 0 |
| 13 | BADARA NOFESSO | F | 30 | 80 | 70 | 8 | 4 |
| 14 | BADARA NOFESSO | M | 13 | 2 | 2 | 0 | 2 |
| 15 | BADARA NOFESSO | F | 10 | 5 | 1 | 3 | 1 |
| 16 | BADARA NOFESSO | F | 7 | 1 | 1 | 0 | 0 |
| 17 | BADARA NOFESSO | M | 5 | 1 | 1 | 0 | 1 |
| 18 | BADARA NOFESSO | F | 5 | 2 | 3 | 0 | 0 |
| 19 | BADARA NOFESSO | M | 34 | 7 | 3 | 1 | 2 |
| 20 | BADARA NOFESSO | M | 11 | 1 | 1 | 0 | 0 |
| 21 | BADARA NOFESSO | M | 39 | 35 | 30 | 0 | 0 |
| 22 | BADARA NOFESSO | M | 15 | 1 | 1 | 0 | 1 |
| 23 | BADARA NOFESSO | M | 23 | 6 | 4 | 0 | 0 |
| 24 | BADARA NOFESSO | F | 18 | 3 | 1 | 0 | 0 |
| 25 | BADARA NOFESSO | F | 13 | 21 | 30 | 0 | 0 |
| 26 | BADARA NOFESSO | M | 10 | 12 | 13 | 2 | 3 |
| 1 | BOLIBANA | M | 45 | 10 | 5 | 1 | 9 |
| 2 | BOLIBANA | F | 32 | 20 | 50 | 0 | 8 |
| 3 | BOLIBANA | M | 21 | 2 | 3 | 0 | 0 |
| 4 | BOLIBANA | M | 36 | 6 | 0 | 0 | 0 |
| 5 | BOLIBANA | F | 28 | 2 | 4 | 3 | 1 |

| | | | | | | | |
|---|---|---|---|---|---|---|---|
| 6 | BOLIBANA | F | 50 | 7 | 0 | 0 | 0 |
| 7 | BOLIBANA | M | 18 | 7 | 4 | 0 | 0 |
| 8 | BOLIBANA | F | 45 | 30 | 50 | 0 | 0 |
| 9 | BOLIBANA | F | 40 | 25 | 50 | 0 | 2 |
| 10 | BOLIBANA | M | 22 | 0 | 5 | 0 | 2 |
| 11 | BOLIBANA | M | 20 | 0 | 1 | 0 | 0 |
| 12 | BOLIBANA | M | 11 | 1 | 7 | 0 | 2 |
| 13 | BOLIBANA | F | 35 | 70 | 60 | 2 | 7 |
| 14 | BOLIBANA | M | 57 | 100 | 110 | 0 | 0 |
| 15 | BOLIBANA | M | 69 | 37 | 14 | 1 | 0 |
| 16 | BOLIBANA | M | 20 | 10 | 10 | 1 | 4 |
| 17 | BOLIBANA | M | 56 | 17 | 3 | 1 | 1 |
| 18 | BOLIBANA | F | 56 | 2 | 9 | 0 | 0 |
| 19 | BOLIBANA | M | 15 | 16 | 1 | 1 | 0 |
| 20 | BOLIBANA | F | 38 | 5 | 2 | 0 | 23 |
| 21 | BOLIBANA | M | 15 | 1 | 3 | 1 | 0 |
| 22 | BOLIBANA | F | 70 | 56 | 1 | 5 | 0 |
| 23 | BOLIBANA | F | 22 | 16 | 38 | 1 | 2 |
| 24 | BOLIBANA | M | 20 | 11 | 11 | 0 | 0 |
| 25 | BOLIBANA | M | 52 | 1 | 3 | 1 | 0 |
| 26 | BOLIBANA | F | 46 | 0 | 5 | 1 | 0 |
| 27 | BOLIBANA | F | 10 | 130 | 100 | 1 | 7 |
| 28 | BOLIBANA | F | 7 | 50 | 10 | 0 | 0 |
| 29 | BOLIBANA | M | 5 | 15 | 10 | 0 | 36 |
| 30 | BOLIBANA | F | 38 | 14 | 2 | 0 | 1 |
| 31 | BOLIBANA | F | 8 | 0 | 1 | 0 | 1 |
| 32 | BOLIBANA | M | 9 | 2 | 0 | 1 | 0 |
| 33 | BOLIBANA | F | 55 | 0 | 4 | 0 | 0 |
| 34 | BOLIBANA | F | 8 | 3 | 1 | 0 | 0 |
| 35 | BOLIBANA | F | 15 | 2 | 4 | 0 | 0 |
| 36 | BOLIBANA | F | 6 | 10 | 20 | 0 | 0 |
| 37 | BOLIBANA | M | 17 | 100 | 60 | 29 | 23 |

| | | | | | | | | |
|---|---|---|---|---|---|---|---|---|
| 38 | BOLIBANA | F | 55 | 56 | 71 | 0 | 1 |
| 39 | BOLIBANA | M | 10 | 9 | 0 | 0 | 0 |
| 40 | BOLIBANA | F | 8 | 1 | 4 | 1 | 0 |
| 41 | BOLIBANA | F | 5 | 3 | 18 | 0 | 0 |
| 42 | BOLIBANA | F | 16 | 5 | 20 | 0 | 8 |
| 43 | BOLIBANA | F | 10 | 10 | 30 | 0 | 0 |
| 44 | BOLIBANA | M | 14 | 4 | 10 | 0 | 1 |
| 45 | BOLIBANA | M | 5 | 10 | 30 | 0 | 0 |
| 46 | BOLIBANA | F | 17 | 1 | 6 | 0 | 0 |
| 47 | BOLIBANA | M | 23 | 12 | 13 | 0 | 0 |
| 1 | CONGALA 2 | M | 20 | 15 | 12 | 0 | 0 |
| 2 | CONGALA 2 | F | 17 | 7 | 6 | 0 | 0 |
| 3 | CONGALA 2 | M | 32 | 5 | 6 | 0 | 0 |
| 4 | CONGALA 2 | F | 26 | 3 | 3 | 12 | 0 |
| 5 | CONGALA 2 | M | 42 | 16 | 3 | 7 | 3 |
| 6 | CONGALA 2 | M | 14 | 9 | 8 | 0 | 1 |
| 7 | CONGALA 2 | F | 8 | 10 | 7 | 0 | 0 |
| 8 | CONGALA 2 | M | 38 | 5 | 3 | 0 | 0 |
| 9 | CONGALA 2 | F | 30 | 25 | 15 | 0 | 0 |
| 10 | CONGALA 2 | M | 44 | 10 | 17 | 1 | 2 |
| 11 | CONGALA 2 | F | 15 | 7 | 3 | 0 | 0 |
| 12 | CONGALA 2 | F | 14 | 10 | 13 | 0 | 0 |
| 13 | CONGALA 2 | F | 32 | 3 | 15 | 6 | 15 |
| 14 | CONGALA 2 | F | 13 | 5 | 20 | 0 | 0 |
| 15 | CONGALA 2 | F | 6 | 10 | 3 | 0 | 0 |
| 16 | CONGALA 2 | M | 33 | 35 | 25 | 0 | 2 |
| 17 | CONGALA 2 | F | 24 | 40 | 32 | 2 | 0 |
| 18 | CONGALA 2 | M | 34 | 5 | 3 | 12 | 9 |
| 19 | CONGALA 2 | M | 45 | 8 | 6 | 0 | 0 |
| 20 | CONGALA 2 | F | 35 | 7 | 5 | 0 | 1 |
| 21 | CONGALA 2 | M | 7 | 10 | 22 | 0 | 1 |
| 22 | CONGALA 2 | M | 41 | 0 | 1 | 0 | 1 |

| | | | | | | | |
|---|---|---|---|---|---|---|---|
| 23 | CONGALA 2 | M | 65 | 17 | 18 | 0 | 0 |
| 24 | CONGALA 2 | M | 29 | 25 | 32 | 0 | 1 |
| 25 | CONGALA 2 | M | 54 | 1 | 2 | 0 | 0 |
| 26 | CONGALA 2 | M | 33 | 12 | 10 | 0 | 0 |
| 1 | KOSSOUMANI | F | 27 | 0 | 1 | 0 | 0 |
| 2 | KOSSOUMANI | F | 40 | 12 | 2 | 0 | 0 |
| 3 | KOSSOUMANI | M | 11 | 0 | 3 | 0 | 0 |
| 4 | KOSSOUMANI | M | 28 | 0 | 1 | 0 | 0 |
| 5 | KOSSOUMANI | M | 25 | 1 | 0 | 0 | 0 |
| 6 | KOSSOUMANI | M | 57 | 4 | 1 | 1 | 0 |
| 7 | KOSSOUMANI | F | 47 | 4 | 2 | 0 | 0 |
| 8 | KOSSOUMANI | M | 8 | 2 | 0 | 0 | 0 |
| 9 | KOSSOUMANI | F | 30 | 1 | 0 | 0 | 0 |
| 10 | KOSSOUMANI | M | 7 | 7 | 5 | 0 | 0 |
| 11 | KOSSOUMANI | F | 8 | 0 | 1 | 0 | 0 |

**Notes:** ICL=Iliac Crest Left; ICR=Iliac Crest Right; mfs/b=number of microfilariae per biopsy
