# Supplementary material for: The impact of ivermectin on onchocerciasis in villages co-endemic for lymphatic filariasis in an area of onchocerciasis recrudescence in Burkina Faso
Source: PLoS Negl Trop Dis. 2021 Mar 1;15(3):e0009117. doi: 10.1371/journal.pntd.0009117 (PMC7920372; doi:10.1371/journal.pntd.0009117)
Supplement: S2 Table — (PDF) [file pntd.0009117.s003.pdf]

SUPPORTING INFORMATION

Nikiema et al. The impact of ivermectin in an area of onchocerciasis recrudescence.

S2 Table.

Numbers of microfilariae for each iliac crest (mfs/b) in the 101 people who were skin snip positive in 2010 but failed to complete the cohort requirements to participate in the skin snip assessment in 2012.

	VILLAGE	Sex	Age	2010 ICL mfs/b	2010 ICR mfs/b
1	BADARA KARABORO	F	31	10	8
2	BADARA KARABORO	F	70	5	2
3	BADARA KARABORO	M	28	40	12
4	BADARA KARABORO	M	13	0	1
5	BADARA KARABORO	F	10	33	10
6	BADARA KARABORO	M	36	1	3
7	BADARA KARABORO	F	26	1	2
8	BADARA KARABORO	F	10	0	3
9	BADARA KARABORO	F	45	1	7
10	BADARA KARABORO	M	8	33	17
11	BADARA KARABORO	F	28	2	1
12	BADARA KARABORO	F	6	7	12
13	BADARA KARABORO	F	10	25	7
14	BADARA KARABORO	F	8	33	22
15	BADARA KARABORO	F	28	2	12
1	BADARA NOFESSO	F	30	3	8
2	BADARA NOFESSO	M	9	15	17
3	BADARA NOFESSO	F	55	3	1
4	BADARA NOFESSO	M	6	7	12
5	BADARA NOFESSO	M	42	85	60
6	BADARA NOFESSO	F	33	15	12
7	BADARA NOFESSO	M	13	5	3
8	BADARA NOFESSO	M	32	15	17
9	BADARA NOFESSO	F	7	3	6
10	BADARA NOFESSO	M	14	5	2
11	BADARA NOFESSO	F	36	25	15
12	BADARA NOFESSO	M	15	1	3
13	BADARA NOFESSO	F	13	3	8
14	BADARA NOFESSO	F	28	7	2
15	BADARA NOFESSO	M	5	3	3

1	BOLIBANA	F	13	0	1
2	BOLIBANA	F	25	1	6
3	BOLIBANA	F	27	2	4
4	BOLIBANA	F	6	2	0
5	BOLIBANA	M	9	2	14
6	BOLIBANA	M	18	51	47
7	BOLIBANA	F	18	15	20
8	BOLIBANA	M	28	2	1
9	BOLIBANA	F	20	2	1
10	BOLIBANA	M	26	2	1
11	BOLIBANA	F	25	0	1
12	BOLIBANA	F	64	3	1
13	BOLIBANA	F	27	0	6
14	BOLIBANA	F	27	3	2
15	BOLIBANA	M	18	2	10
16	BOLIBANA	M	28	2	1
17	BOLIBANA	M	26	2	13
18	BOLIBANA	M	14	2	1
19	BOLIBANA	M	25	5	1
20	BOLIBANA	M	38	12	15
21	BOLIBANA	F	36	6	8
22	BOLIBANA	F	16	11	23
23	BOLIBANA	F	24	5	16
24	BOLIBANA	M	18	12	44
25	BOLIBANA	F	10	15	2
26	BOLIBANA	F	56	30	10
27	BOLIBANA	M	50	5	8
28	BOLIBANA	F	14	6	5
29	BOLIBANA	F	5	26	11
30	BOLIBANA	F	70	0	1
31	BOLIBANA	M	45	10	5
32	BOLIBANA	F	37	1	0
33	BOLIBANA	F	14	0	1
34	BOLIBANA	M	36	1	0
35	BOLIBANA	M	26	3	4
36	BOLIBANA	M	40	9	15
37	BOLIBANA	M	16	34	28
38	BOLIBANA	M	16	1	0
39	BOLIBANA	F	55	6	6
40	BOLIBANA	M	35	1	0
41	BOLIBANA	F	20	12	9
42	BOLIBANA	M	19	1	0

43	BOLIBANA	M	70	12	25
44	BOLIBANA	F	69	12	0
45	BOLIBANA	M	21	20	15
46	BOLIBANA	F	26	18	21
47	BOLIBANA	F	29	3	0
48	BOLIBANA	M	33	6	12
49	BOLIBANA	F	55	2	14
50	BOLIBANA	F	16	2	3
51	BOLIBANA	F	35	7	12
52	BOLIBANA	M	13	4	2
53	BOLIBANA	M	13	2	5
54	BOLIBANA	M	8	0	4
55	BOLIBANA	F	25	4	2
56	BOLIBANA	M	27	6	10
57	BOLIBANA	M	18	3	0
58	BOLIBANA	F	54	3	0
59	BOLIBANA	F	22	4	6
60	BOLIBANA	M	55	47	65
61	BOLIBANA	M	47	10	15
62	BOLIBANA	F	34	2	6
1	CONGALA 2	M	12	0	3
2	CONGALA 2	F	25	8	14
3	CONGALA 2	M	15	1	1
1	KOSSOUMANI	M	75	2	0
2	KOSSOUMANI	M	69	5	7
3	KOSSOUMANI	M	8	0	1
4	KOSSOUMANI	F	8	4	1
5	KOSSOUMANI	F	33	1	1
6	KOSSOUMANI	F	13	2	3
