# Supplementary material for: The impact of ivermectin on onchocerciasis in villages co-endemic for lymphatic filariasis in an area of onchocerciasis recrudescence in Burkina Faso
Source: PLoS Negl Trop Dis. 2021 Mar 1;15(3):e0009117. doi: 10.1371/journal.pntd.0009117 (PMC7920372; doi:10.1371/journal.pntd.0009117)
Supplement: S3 Table — (PDF) [file pntd.0009117.s004.pdf]

SUPPORTING INFORMATION

Nikiema et al. The impact of ivermectin in an area of onchocerciasis recrudescence.

S3 Table.

Therapeutic Coverage of mass drug administration (MDA) with ivermectin against lymphatic filariasis in the onchocerciasis recrudescence area of Cascades Region in Burkina Faso up to 2011 when ivermectin was also being administered against onchocerciasis. Note that the figures for 2004 to 2007 exclude Mangodara District in which MDA was introduced in 2008.

Year	Mean Therapeutic Coverage (%)
2004	79.03*
2005	78.22*
2006	81.47*
2007	79.44*
2008	85.02
2009	79.33
2010	89.94

*Figure does not include Mangodara District where geographic and therapeutic coverage was 0%.

In the onchocerciasis recrudescence area, geographic coverage and mean therapeutic coverage both reached 100% and 80% (respectively) in only two out of seven years.
